# Significant decrease of maternal mitochondria carryover using optimized spindle-chromosomal complex transfer

**DOI:** 10.1371/journal.pbio.3002313

**Published:** 2023-10-05

**Authors:** Xiaoyu Liao, Wenzhi Li, Kaibo Lin, Wei Jin, Shaozhen Zhang, Yao Wang, Meng Ma, Yating Xie, Weina Yu, Zhiguang Yan, Hongyuan Gao, Leiwen Zhao, Jiqiang Si, Yun Wang, Jiaying Lin, Chen Chen, Li Chen, Yanping Kuang, Qifeng Lyu

**Affiliations:** Department of Assisted Reproduction, Shanghai Ninth People’s Hospital, Shanghai JiaoTong University School of Medicine, Shanghai, People’s Republic of China; Institute for Ageing and Health, UNITED KINGDOM

## Abstract

Mutations in mitochondrial DNA (mtDNA) contribute to a variety of serious multi-organ human diseases, which are strictly inherited from the maternal germline. However, there is currently no curative treatment. Attention has been focused on preventing the transmission of mitochondrial diseases through mitochondrial replacement (MR) therapy, but levels of mutant mtDNA can often unexpectedly undergo significant changes known as mitochondrial genetic drift. Here, we proposed a novel strategy to perform spindle-chromosomal complex transfer (SCCT) with maximal residue removal (MRR) in metaphase II (MII) oocytes, thus hopefully eliminated the transmission of mtDNA diseases. With the MRR procedure, we initially investigated the proportions of mtDNA copy numbers in isolated karyoplasts to those of individual oocytes. Spindle-chromosomal morphology and copy number variation (CNV) analysis also confirmed the safety of this method. Then, we reconstructed oocytes by MRR-SCCT, which well developed to blastocysts with minimal mtDNA residue and normal chromosomal copy numbers. Meanwhile, we optimized the manipulation order between intracytoplasmic sperm injection (ICSI) and SCC transfer and concluded that ICSI-then-transfer was conducive to avoid premature activation of reconstructed oocytes in favor of normal fertilization. Offspring of mice generated by embryos transplantation in vivo and embryonic stem cells derivation further presented evidences for competitive development competence and stable mtDNA carryover without genetic drift. Importantly, we also successfully accomplished SCCT in human MII oocytes resulting in tiny mtDNA residue and excellent embryo development through MRR manipulation. Taken together, our preclinical mouse and human models of the MRR-SCCT strategy not only demonstrated efficient residue removal but also high compatibility with normal embryo development, thus could potentially be served as a feasible clinical treatment to prevent the transmission of inherited mtDNA diseases.

## Abstract 2

Mutations in mitochondrial DNA (mtDNA) cause a variety of serious multi-organ human diseases, but existing means of preventing mtDNA transmission are problematic. This study presents a novel technically optimized strategy to perform spindle-chromosomal complex transfer (SCCT) with minimal mtDNA carryover and excellent embryo development in both mouse and human models.

## Introduction

Mitochondria are essential intracellular organelles that supply energy in the form of adenosine triphosphate generated via oxidative phosphorylation and contain their own double-stranded circular DNA. Although the mitochondrial genome only contains 37 genes, mutations may occur in certain mitochondrial DNA (mtDNA). If mutant mtDNA is present in maternal oocytes, it can be inherited to future generations [1–2]. In some cases, even if the initial proportion of mutant mtDNA is low, levels may unpredictably change during development due to mitochondrial genetic drift [3–4]. When heteroplasmic mtDNA proliferate beyond normal ranges, mutation-related diseases may occur, leading to clinical symptoms such as deafness, blindness, diabetes, muscle weakness, and liver failure at any age [5–7]. Treatment options are generally limited for those suffering from these illnesses, so interventions that prevent the transmission of maternally inherited mitochondrial diseases to offspring are encouraged. Promising approaches include mitochondrial replacement (MR) between gametes or embryos, such as pronuclear transfer, poly body transfer, and spindle transfer [8–10].

Proposed as one of classic MR approaches, spindle-chromosomal complex transfer (SCCT) utilizes micromanipulation techniques in MII-stage oocytes to transfer the nuclear genetic material, specifically the spindle with maternally derived chromosomes attached, from one unfertilized oocyte to another that has had its own nuclear material removed. The resulting oocyte is then fertilized to enable embryo development. In 2009, Tachibana and colleagues successfully performed SCCT in rhesus monkeys without affecting subsequent fertilization and developmental competence, resulting in the birth of healthy offspring [11]. In 2013, researchers subsequently reported that mtDNA transferred with spindles was detected at levels below 1%, or technically undetectable, in human blastocysts and embryonic stem cell (ESC) lines [12–13]. In 2017, John Zhang and his team successfully achieved the first SCCT three-parent baby with human oocytes in Mexico [14]. Meanwhile, it was reported that heteroplasmic mtDNA increased from 1.3% at derivation to 53.2% at passage 36 in 1 human ESC line [15]. Additionally, Kang and colleagues identified that 1 ESC line exhibited a gradual increase in maternal mtDNA from 19% at passage 2 to 100% at passage10 in a Leigh syndrome patient in 2016 [16]. These findings suggested that mitochondrial genetic drift occurred with current SCCT technology. This might be due to the fact that during micromanipulation, SCCs in oocytes were inevitably isolated together with some cytoplasm containing a portion of mitochondria, which eventually resulted in the transfer of heteroplasmic mtDNA from patients’ oocytes to those of recipients [17]. Therefore, SCC-reconstructed oocytes might have a significant amount of heteroplasmic mtDNA, and the risk of genetic drift would increase with the amount of mitochondria carried over, making patients more susceptible to mtDNA diseases later on.

Based on these considerations, we believed that actively pursuing extremely low or negligible mtDNA carryover during SCCT micromanipulation could be achieved, which would eliminate the risk of genetic drift and clinically prevent the transmission of mtDNA diseases. For this purpose, here we presented a novel strategy named maximal residue removal (MRR) to extremely remove transferred mtDNA along with spindles in mouse and human MII oocytes, by which SCCs were sucked using biopsy micropipettes of a finer inner diameter (ID), and then the carried cytoplasm surrounding SCCs was further swung away in a special polyvinylpyrrolidone (PVP) solution-based mixture. Initially, we evaluated the effectiveness and safety of this MRR procedure by analyzing mtDNA copy numbers and genetic copy number variation (CNV) in each individual SCC. Then, we reconstructed oocytes between mice with different mtDNA origins and investigated developmental outcomes following fertilization by intracytoplasmic sperm injection (ICSI), mtDNA heteroplasmy levels, and chromosomal copy numbers in SCCT blastocysts. During the SCCT, we observed and overcame the transfer-triggered premature activation of reconstructed oocytes by adjusting the manipulation order between ICSI and SCC transfer, simultaneously resulting in an improvement in normal fertilization rates, a shorter procedural duration and the potential avoidance of oocyte sacrifices induced by the transfer-then-ICSI method. Oviduct transplantation in vivo and ESCs derivation in vitro were also conducted to confirm the feasibility of MRR approach in mouse SCCT embryos. Ultimately, we successfully achieved minimal mtDNA carryover and excellent development of SCCT embryos employing the MRR procedure in human MII oocytes.

## Materials and procedures

### Ethics statement

In the mouse model, our experiments were conducted in accordance with the Guide for Care and Use of Animals for Research Purposes and approved by the Animal Experimental Ethics Committee of Shanghai Ninth People’s Hospital affiliated to Shanghai JiaoTong University School of Medicine (approval number: HKDL [2018] 228).

Human gamete manipulations were permitted by the Independent Ethics Committee of Shanghai Ninth People’s Hospital affiliated to Shanghai JiaoTong University School of Medicine (approval number: SH9H-2018-T57-1). Oocytes and sperm were donated voluntarily for this study from participants, who had signed an informed consent stating that oocytes would be fertilized to create embryos for research purposes, and would not be used for reproductive transplantation in vivo. Egg donors were recruited from women aged younger than 40 years old, who were undergoing infertility treatment and retrieved abundant mature eggs (more than 20 per ICSI cycle) for donating 3 to 5 eggs in each cycle in the Assisted Reproduction Department of our hospital. And they would receive a deduction in the ICSI treatment fee and no extra financial compensation.

### Gamete preparation and processing

Oocytes and sperm were retrieved and processed as described previously [18]. In brief, wild-type ICR and C57BL/6 female mice (4 to 6 weeks old) were intraperitoneally injected pregnant mare serum gonadotrophin and human chorionic gonadotrophin (PMSG and hCG, 10 IU, Ningbo No. 2 Hormone Factory, China) at a 48 h interval to induce superovulation. Then, 16 to 18 h later, mice were euthanized by cervical dislocation, and cumulus-oocyte complexes (COCs) were recovered in modified human tubal fluid medium (mHTF, 90126, IrvineScientific, United States of America) supplemented with 10% serum substitute supplement (SSS, 99193, IrvineScientific) at 37°C. The surrounding cumulus cells were denuded through a brief exposure to 5 μg/mL hyaluronidase (H4272, SIGMA, Germany). Then, oocytes were adequately rinsed with fresh mHTF medium and subsequently cultured in G1 PLUS medium (10128, Vitrolife, Sweden) covered with mineral oil (ART-4008-5P, SAGE, USA) at 37°C in 6% CO_2_ and 5% O_2_ for 0.5 to 1 h before micromanipulations. Mouse sperm was collected from the cauda epididymis and vas deferens of wild-type C57BL/6 male mice (8 to 10 weeks old), and swim-up for at least 30 min in mHTF medium was conducted to acquire well-motile sperm.

For human oocytes collection, controlled ovarian stimulation was performed. And ovulation was triggered by injecting 2,000 IU of hCG (Lizhu Pharmaceutical Trading Co., China), followed by ultrasound-guided follicles aspiration approximately 36 h later. Then, the retrieved COCs were treated with hyaluronidase to disaggregate cumulus and granulosa cells, and oocytes were isolated and placed in G1 PLUS medium. Human semen was collected by masturbatory ejaculation and the supernatant containing motile sperm cells was purified by gradient centrifugation.

### Spindle-chromosomal complex transfer

**Fig 1:**
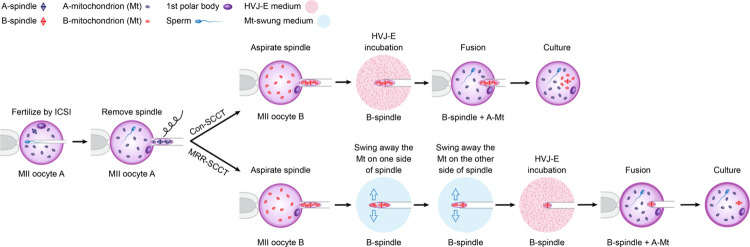
A novel SCCT procedure for MRR of mtDNA. a: Perform ICSI on the cytoplasmic-recipient MII oocyte (A); b: remove the SCC of oocyte-A; c: aspirate the SCC of karyoplast-donor oocyte (B) into micropipette with a much finer ID; d: swing away the carried cytoplasm surrounding SCC in a special PVP-based medium; e: expose the SCC karyoplast-B to HVJ-E protein briefly; f: fusion with the fertilized and enucleated oocyte-A; g: culture in medium. ICSI, intracytoplasmic sperm injection; ID, inner diameter; MRR, maximal residue removal; mtDNA, mitochondrial DNA; PVP, polyvinylpyrrolidone; SCCT, spindle-chromosomal complex transfer.

For SCCT micromanipulation, mature MII oocytes were identified by the presence of the first polar body (PB1) and subjected to our innovative MRR procedure as demonstrated in the diagram (Fig 1) and video (S1 Video).

Firstly, cytoplasmic-recipient oocytes were fertilized by ICSI, and the optimized ICSI technique previously described was employed for mouse oocytes [19]. Then, the recipient oocytes were immediately transferred to 2.5 μm cytochalasin B (CB, 14930-96-2, SIGMA) medium covered with mineral oil in a glass-bottomed dish and incubated for 5 to 10 min. This dish was placed on the heating plate of an inverted microscope (IX73, Olympus, Japan) equipped with a micromanipulation system (Integra 3, RI) and the Oosight Imaging System (Hamilton Thorne, USA) for spindle visualization.

We situated the spindle of the recipient oocyte above at the 3 o’clock position and secured it with a holding micropipette (MPH-MED-35, ORIGIO, Denmark). An enucleation micropipette with an ID of 15 μm (SZD-15-35, Sunlight Medical, USA) was inserted through a small opening in the zona pellucida drilled with a low-intensity laser (ZILOS-tk, Hamilton Thorne), and the SCC was removed by aspirating it into the micropipette, which was surrounded by certain amounts of cytoplasm and enveloped with the plasma membrane (karyoplast). SCCs in spindle-donor oocytes were isolated in the same way. Specifically in the MRR procedure, the ID of micropipette used was 12 μm for mouse oocytes and 10 μm for human oocytes, respectively. And the donor karyoplasts were subsequently transferred into a PVP-based mixture consisting of 10% PVP (Diagens Biotechnology Co., China), SSS, and 2.5 μm CB with 5:3:2 ratio. Then, the residual cytoplasm on the left and right sides of spindle in a karyoplast was pushed out of the micropipette in turn, exposed to the mixture, and carefully swung away while still enveloped in an intact plasma membrane.

Subsequently, fusion was mainly performed as previously described [18]. In short, half of a donor karyoplast was briefly exposed to HVJ-E protein (ISK, Japan) for 40 to 60 s, placed within the perivitelline space of a fertilized and enucleated recipient oocyte, and pressed tightly in contact with the oolemma for 3 to 5 min. When membrane fusion occurred, reconstructed oocytes were rinsed with fresh mHTF medium and cultured in KSOM (MR-107, Millipore, Germany) or G1 PLUS medium. The KSOM medium for mouse oocytes was changed every other day, and human oocytes in G1 PLUS medium would be transferred to G2 PLUS medium (10132, Vitrolife) on the third day after fertilization for consecutive culture until the sixth day.

### Oocyte immunofluorescence assay

Reconstructed oocytes of mice were fixed with 2% paraformaldehyde at 4°C overnight. Then, the oocytes were permeabilized with 0.25% Triton X-100 at room temperature for 30 min, washed with 1% bovine serum albumin (BSA) in PBS for 3 times every 5 min and blocked with 1% BSA for 30 min. Then, the microtubules of spindles were labeled with anti-α tubulin (1:200 diluted, ab195887, Abcam) by incubating at 4°C overnight and avoiding light. After washing the samples, we stained nucleus with 4′,6-diamidino-2-phenylindole (DAPI) for 10 min at room temperature. Finally, the samples were washed thoroughly again and then observed under a laser scanning confocal microscope (Olympus).

### CNV analysis in karyoplasts and blastocysts

For chromosomal CNV identification, SCC karyoplasts were transferred into the commercial sample preservation solution with a micropipette under the inverted microscope. The zona pellucida of blastocytes was pre-removed by digesting with acid tyrode’s solution (R23013, Yuanye Bio-Technology Co., China), and then blastocytes were placed into the preservation solution and stored at −20°C. CNV analysis of karyoplasts and blastocysts based on the next generation sequencing was carried out uniformly by the Yikon Genomics (China) company.

### MtDNA copy number detecting by droplet digital PCR (ddPCR)

For mtDNA extraction, samples were lysed with 50 mM NaOH (4.5 μL for karyoplasts, 8.5 μL for oocytes and blastocytes, and 17.0 μL for ESCs, respectively) for 30 min at 95°C and neutralized with 1.0 M Tris-HCl (pH 8.0; 0.5 μL for karyoplasts, 1.5 μL for oocytes and blastocytes, and 3.0 μL for ESCs, respectively). To detect heteroplasmy levels, specific nucleotides probes (S1 Table) for mouse mtDNA sequence variant (m. 9461C > T) in ND3 gene were designed and synthesized by the Integrated DNA Technologies (Singapore) company. A ddPCR supermix (1863025, Bio-Rad Laboratories, USA) for probes was used for each samples, mixed with 900 nM primers (S1 Table), 250 nM probes, and 1.0 μL DNA template. The length of amplification products was 85 base pairs. Droplets were generated by the QX200 Droplet Generator, then transferred into 96-well plates, sealed with Pierceable Foil Heat Seal and cycled in the C1000 Thermal Cycle. PCR reactions were performed as follows: 95°C, 10 min for 1 cycle; 94°C, 30 s followed by 60°C, 1 min for 40 cycles; 98°C, 10 min for 1 cycle. Droplets were read by the QX200 Droplet Reader and then data were analyzed using the QuantaSoft Software (Bio-Rad Laboratories).

### Mouse ESCs derivation and subculture

To derivate ESCs, mouse blastocysts were treated with acid tyrode’s solution to remove the zona pellucida, and the inner cell masses of blastocysts were isolated following by planting onto a CF1-feeder layer in B-27 Plus medium (A3653401, Gibco, USA) at 37°C in 6% CO_2_ and 5% O_2_. Cell outgrowths were manually dissociated into small clumps with a microscalpel and replated on fresh layers [20]. After the initial passage, colonies with ESC-like morphology were selected for further propagation, and culture medium was changed daily [21]. ESC colonies typically split every 5 to 7 days and were subcultured in fresh medium without feeder. For detecting mtDNA residues in ESCs by ddPCR, we randomly picked out 5 to 10 clones in each cell lines every 3 passages.

### Mouse embryo transplantation in vivo

Healthy female ICR mice (8 to 12 weeks old) were caged with ligated male mice. In the next morning, ICR mice with vaginal plug were picked out and anesthetized with isoflurane. Then, the reconstructed embryos together with non-manipulated control embryos at 2-cell stage were surgically transferred into the fimbria of fallopian tube in the pseudopregnant ICR mice, which were fed with sufficient water and high fat food. Approximately 21 to 23 days later, cubs of F1 generation were born, and body weights were measured weekly for 2 months. When growing up until puberty, female offspring were mated with wild-type C57 males, resulting in the birth of F2 cubs. Toes of F1 and F2 pups were respectively biopsied for testing mtDNA residues by ddPCR.

### Statistical analysis

Statistical analysis was performed using Prism 9.0 statistical analysis program (GraphPad). Results were showed as mean ± standard error of mean (X ± SEM). Comparisons between 2 groups were performed with *t* test, and chi-square test or one-way ANOVA with LSD test was used for comparing 3 or more groups. The statistical significance was set at *p* < 0.05 (* denoted *p* < 0.05, ** denoted *p* < 0.01, *** denoted *p* < 0.001, and ****denoted *p* < 0.0001).

## Results

### Maximal residue removal of mtDNA with a novel SCCT procedure

**Fig 2:**
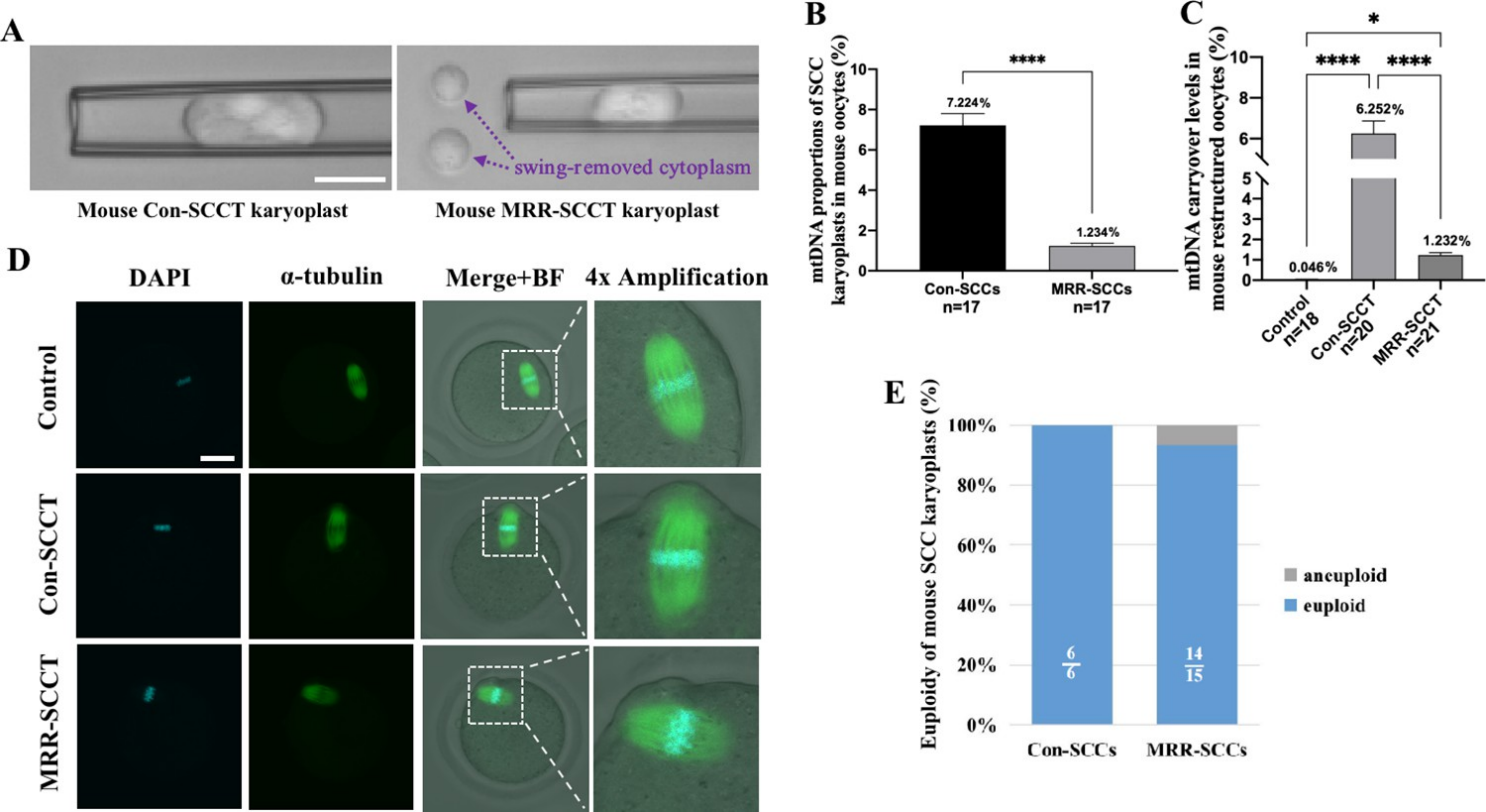
MtDNA carryover removal and SCC integraty by MRR manipulation in mouse oocytes. (A) Representative images of mouse Con-SCCT and MRR-SCCT karyoplasts isolated by micropipettes with ID of 15 μm and 12 μm, respectively. The purple arrows indicated the swing-removed cytoplasm around spindle. Scale bars, 20 μm. (B) MtDNA proportions of SCC karyoplasts in corresponding enucleated ICR oocytes (mtDNA copy number of karyoplast/total mtDNA copy numbers of karyoplast and enucleated oocyte, %, Y-axis) in the Con-SCCT (*n* = 17) and MRR-SCCT (*n* = 17) groups (X-axis). The error bars represented SEM with mean values shown in each group. The whiskers indicated a significant difference between groups (**** denoted *P* < 0.0001). (C) MtDNA carryover levels in mouse reconstructed oocytes (karyoplast and cytoplasm were from ICR and C57BL/6 mice, respectively, %, Y-axis) in the control (*n* = 18), Con-SCCT (*n* = 20), and MRR-SCCT (*n* = 21) groups (X-axis). * Denoted *p* < 0.05 and **** denoted *P* < 0.0001. (D) Morphological analysis of spindle and nuclear DNA by immunofluorescence labeling with antibody to α-tubulin (green) and DAPI (blue) in the control, Con-SCCT, and MRR-SCCT mouse oocytes. BF referred to bright field. Images on the right were higher magnification views of whitely boxed areas in the left. Scale bars, 50 μm. (E) Euploidy of mouse Con-SCC and MRR-SCC karyoplasts. Proportions of euploid (blue, white fractional numbers) and aneuploid (gray) chromosomes were shown in both groups. The numerical data were listed in S1 Data. Con-SCCT, conventional SCCT; ID, inner diameter; MRR, maximal residue removal; mtDNA, mitochondrial DNA.

In this study, we performed 3 groups to compare mtDNA residue and embryo development. In the conventional SCCT (Con-SCCT) group, mouse SCCs were aspirated using micropipettes with an ID of 15 μm without cytoplasmic removal (Fig 2A). Whereas for the MRR-SCCT, we utilized micropipettes with an ID of 12 μm to obtain SCC karyoplasts in MII oocytes, following by further cytoplasm-swinging away in a PVP-based mixture (Fig 1, S1 Video, Fig 2A). Besides, non-SCCT manipulated but ICSI-fertilized oocytes were served as the control group. In the mouse SCCT model, we adopted oocytes from ICR mice as the nuclear donor and oocytes from C57BL/6 mice as the cytoplasmic receptor.

Firstly, we respectively quantified mtDNA copy numbers of SCC karyoplasts and corresponding enucleated oocytes from ICR mice by ddPCR, and calculated proportions. Results showed that in the Con-SCC group, mtDNA proportion of a karyoplast absorbed was average 7.224% (*n* = 17) in an oocyte (Fig 2B). Whereas in the MRR-SCC group, this proportion decreased to 1.234% on average (range 0.676% to 2.632%, *n* = 17, Fig 2B). To further investigate the carried mitochondrial quantity, we detected mtDNA heteroplasmy levels in restructured oocytes with ICR and C57BL/6 mice. In general, the heteroplasmy rate of mtDNA was average 1.232% (*n* = 21) through the MRR manipulation, which was significantly lower than that in the Con-SCCT group (6.252% on average, *n* = 20) (*p* < 0.0001) (Fig 2C), suggesting that a significant amount of mitochondria surrounding spindles would be removed with the MRR procedure in restructured oocytes of mice.

As indicated above, it was technically feasible to greatly reduce mitochondrial residue by transferring SCC karyoplasts with minimal cytoplasm, whereas question remained whether the MRR micromanipulation would cause abnormality of SCCs. To address this concern, we stained restructured oocytes of mice with anti-α tubulin antibody and DAPI to visualize the spindle and nuclear DNA via confocal microscopy. Results showed that MRR-SCCs contained intact microtubules and chromosomal structures, which were morphologically similar to those in the Con-SCCT and control groups (Fig 2D). Next, chromosomal CNV analysis of SCC karyoplasts confirmed normal copy numbers of chromosomes in the MRR-SCC and Con-SCC groups. Both had a diploid genome (Fig 2E and S1 Data) and no DNA signal was detected in the swing-removed cytoplasts (*n* = 5, S1 Data). Hence, this novel mitochondrial removal approach of MRR would not compromise the integraty of spindle and chromosome and could be readily performed in nuclear transfer.

### Manipulation order between ICSI and SCC transfer

To shorten procedure duration and avoid potential sacrifice of restructured oocytes by ICSI, we employed an alternative way to introduce sperm into MII oocytes before SCC transfer and found that mouse zygotes displayed regular 2 pronuclei and the second polar body (PB2) at a higher incidence (88.32% on average, *n* = 47) compared to that in the conventional transfer-then-ICSI group (70.30% on average, *n* = 50) (*p* < 0.05) (S1A Fig).

This finding was consistent with previous reports that meiotic spindles might undergo premature activation during the micromanipulation of spindle transfer, leading to an incomplete meiosis resumption and aberrant fertilization subsequently [13]. To further explain the theory, we stained reconstructed oocytes of mice by immunolabeling with anti-α tubulin and DAPI for visualizing spindle morphology and meiotic stage. Results displayed that spindles were present in all the SCC transferred oocytes, and some resumed the meiotic division and progressed to the anaphase of meiosis II before ICSI (S1B Fig). Whereas in the ICSI-then-transfer group, reconstructed oocytes maintained metaphase II stage uniformly (S1B Fig). Thus, these results further indicated that the manipulation of spindle transfer might trigger premature activation of MII oocytes and subsequent resumption of meiosis, which could be favorably avoided by the ICSI-then-transfer approach adopted in our latter experiments.

### Developmental potential of mouse SCC transferred oocytes in vitro

**Fig 3:**
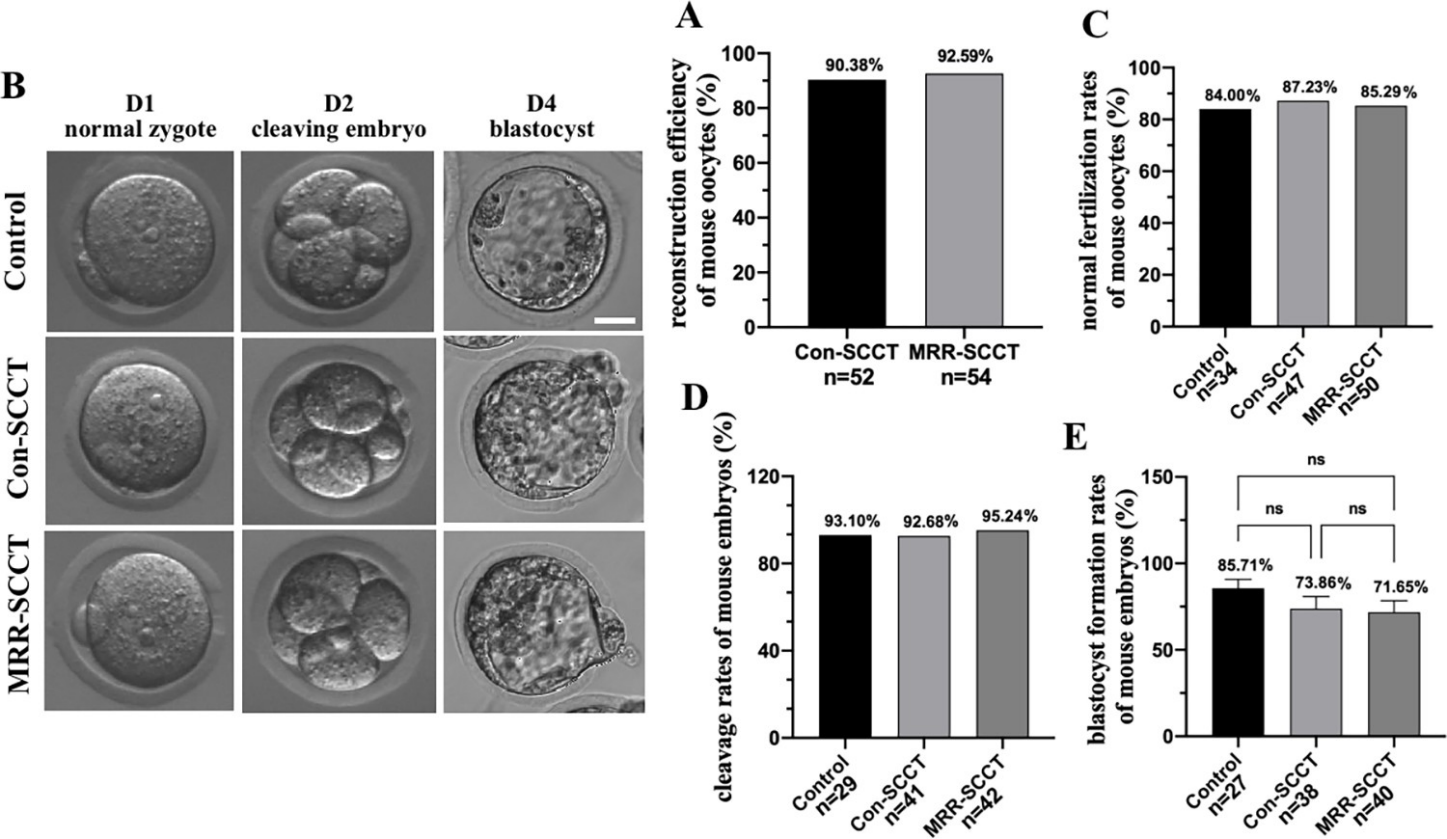
Developmental potential of mouse SCC transferred oocytes in vitro. (A) Reconstruction efficiency of mouse oocytes (%, Y-axis) in the Con-SCCT (*n* = 52) and MRR-SCCT (*n* = 54) groups (X-axis). Mean values were shown above the columns in each group. (B) Morphological images of normal zygotes, cleaving embryos and blastocysts in the control, Con-SCCT and MRR-SCCT groups. The day of fertilization was calculated as Day 0 (D0). Scale bars, 20 μm. (C) Quantification of normal fertilization rates of mouse oocytes (%, Y-axis) in the control (*n* = 34), Con-SCCT (*n* = 47), and MRR-SCCT (*n* = 50) groups (X-axis). (D) Quantification of cleavage rates of mouse embryos (%, Y-axis) in the control (*n* = 29), Con-SCCT (*n* = 41), and MRR-SCCT (*n* = 42) groups (X-axis). (E) Quantification of blastocyst formation rates (%, Y-axis) of mouse embryos in the control (*n* = 27), Con-SCCT (*n* = 38), and MRR-SCCT (*n* = 40) groups (X-axis). The error bars represented SEM with mean values shown in each group. Statistics indicated no significant differences between groups (*p* > 0.05). ns: not significant. The numerical data were listed in S2 Data. Con-SCCT, conventional SCCT; MRR, maximal residue removal; SCCT, spindle-chromosomal complex transfer.

Next, we investigated the safety and efficiency of MRR-SCCT for replacing the mitochondrial genotype in mouse oocytes with different mtDNA origins. SCCs from oocytes of ICR mice were isolated and subsequently fused into the fertilized and enucleated recipient oocytes from C57BL/6 mice using HVJ-E protein (S2 Fig). Karyoplast-cytoplast reconstruction efficiencies including survival and fusion rates were very high in both groups; 47 (90.38%) out of 52 and 50 (92.59%) out of 54 reconstructed oocytes were obtained after the Con-SCCT and MRR-SCCT, respectively (Fig 3A). And normal fertilization rates were calculated in groups; rate of 84.00% (*n* = 50) in the MRR-SCCT group was comparable with those in the Con-SCCT (87.23%, *n* = 47) and control (85.29%, *n* = 34) groups (Fig 3B and 3C). Moreover, the MRR-SCCT-generated embryos cleaved at a frequency of 95.24% (Fig 3B and 3D), and 75.00% on average developed into blastocysts (Fig 3B and 3E), which were comparable to those in the Con-SCCT group (92.68% of embryos cleaved and 73.68% developed to blastocysts) (*p* > 0.05), and were statistically similar to those in the controls (93.10% and 85.19%, respectively) (*p* > 0.05). These implied that SCC transfer in mouse oocytes employing the MRR procedure did not impair subsequent normal fertilization and embryonic developmental competence in vitro.

### MtDNA residual levels and chromosomal copy numbers in mouse SCC transferred blastocysts

**Fig 4:**
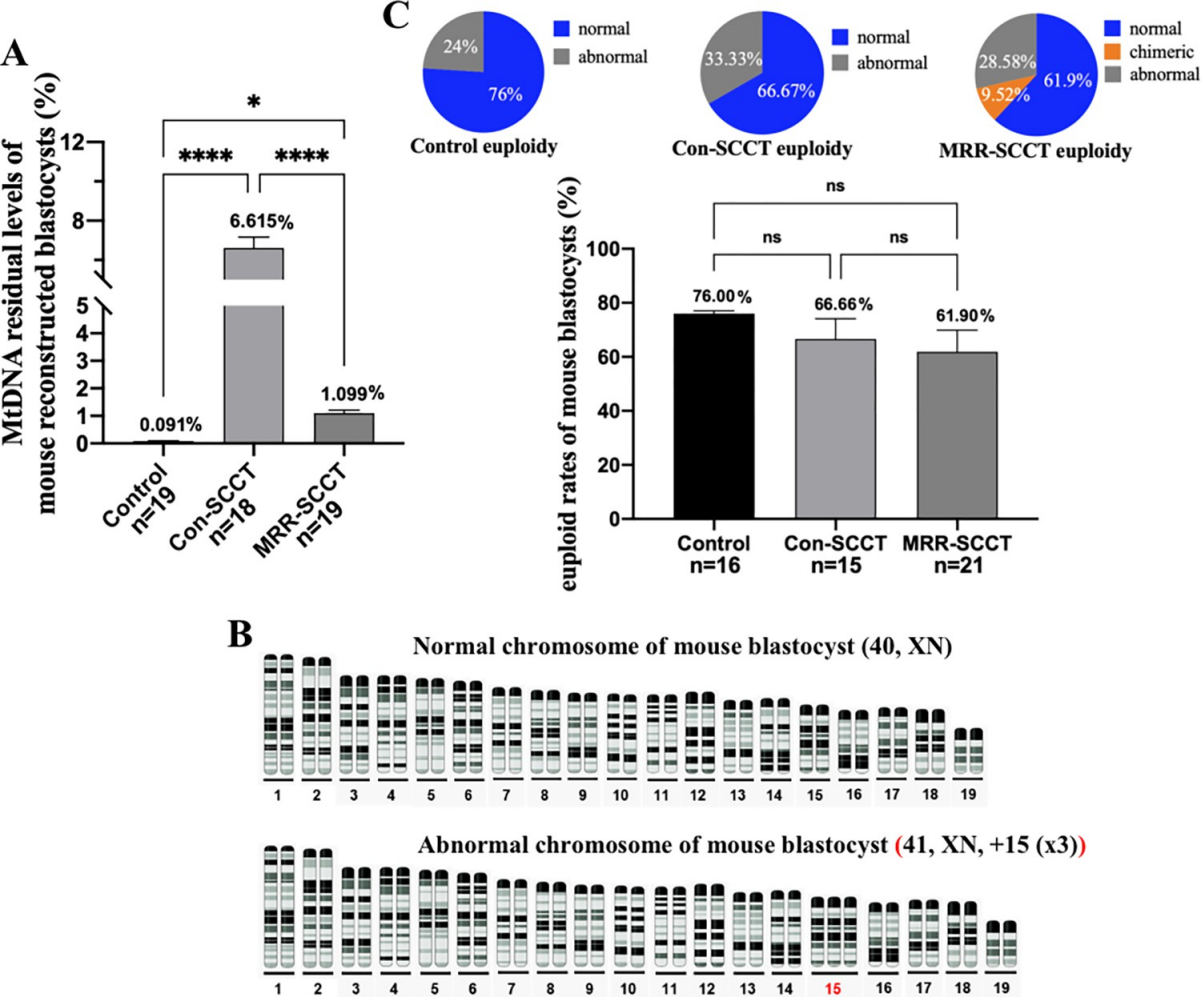
MtDNA residual levels and chromosomal copy numbers in mouse SCC transferred blastocysts. (A) Quantification of mtDNA residual levels in mouse reconstructed blastocysts (karyoplast and cytoplasm were from ICR and C57BL/6 mice, respectively, %, Y-axis) in the control (*n* = 19), Con-SCCT (*n* = 18), and MRR-SCCT (*n* = 19) groups (X-axis). The error bars represented SEM with mean values shown in each group. The whiskers indicated significant differences between groups (* denoted *p* < 0.05 and **** denoted *P* < 0.0001). (B) Representative images of normal and abnormal chromosomes in mouse blastocysts. The numbers under chromosomes showed the sequence of paired chromosomes, and the red number indicated an abnormal chromosome. (C) Quantification of euploid rates in mouse blastocysts (%, Y-axis) in the control (*n* = 16), Con-SCCT (*n* = 15), and MRR-SCCT (*n* = 21) groups (X-axis). Statistics indicated no significant differences between groups (*p* > 0.05, ns: not significant). Proportions (white percentages) of normal (blue), chimeric (orange), and abnormal (gray) chromosomes in each group were respectively shown in the upper pie charts. The numerical data were listed in S3 Data. Con-SCCT, conventional SCCT; MRR, maximal residue removal; mtDNA, mitochondrial DNA; SCCT, spindle-chromosomal complex transfer.

In order to further assess the effectiveness of the MRR-SCCT approach, mtDNA residual rates in mouse blastocysts were examined by ddPCR. Quantitative ddPCR analysis was based on the existing mtDNA sequence difference between mice to distinguish mitochondrial origins in reconstructed blastocysts. In this study, 2 different probes were designed for mtDNA identification between ICR and C57BL/6 mice (S1 Table). Thus, where heteroplasmy was present, the proportion of spindle-donor’s mtDNA would be detected. Tests showed that the heteroplasmy level of MRR-SCCT-produced blastocysts was 1.099% on average (*n* = 19), which was significantly lower than that in the Con-SCCT group (*p* < 0.0001) (Fig 4A). This was consistent with above results that MRR procedure could hugely decrease mtDNA carryovers in mouse SCC karyoplasts and reconstructed oocytes (Fig 2B and 2C).

To determine whether SCCT micromanipulation might induce chromosomal abnormality in blastocysts produced by the novel MRR procedure, we conducted chromosomal CNV identification by the next generation sequencing technology. Expanded mouse blastocysts in the SCCT and corresponding control groups were analyzed. Results revealed that approximately 61.90% (*n* = 21) of MRR-SCCT-derived blastocysts contained normal copy numbers (male-40, XY or female-40, XX) with 9.52% behaving chimeric phenotype (chimeric rate was below 50% in each chimerism), which was comparable to those in the Con-SCCT (*p* = 0.6226) and intact control (*p* = 0.1779) groups (Fig 4B and 4C). These reveled that MRR-SCCT-generated blastocysts of mice maintained a similar euploidy landscape to those produced by the Con-SCCT and controls.

### Mouse SCCT-generated embryo transplantation in vivo

**Fig 5:**
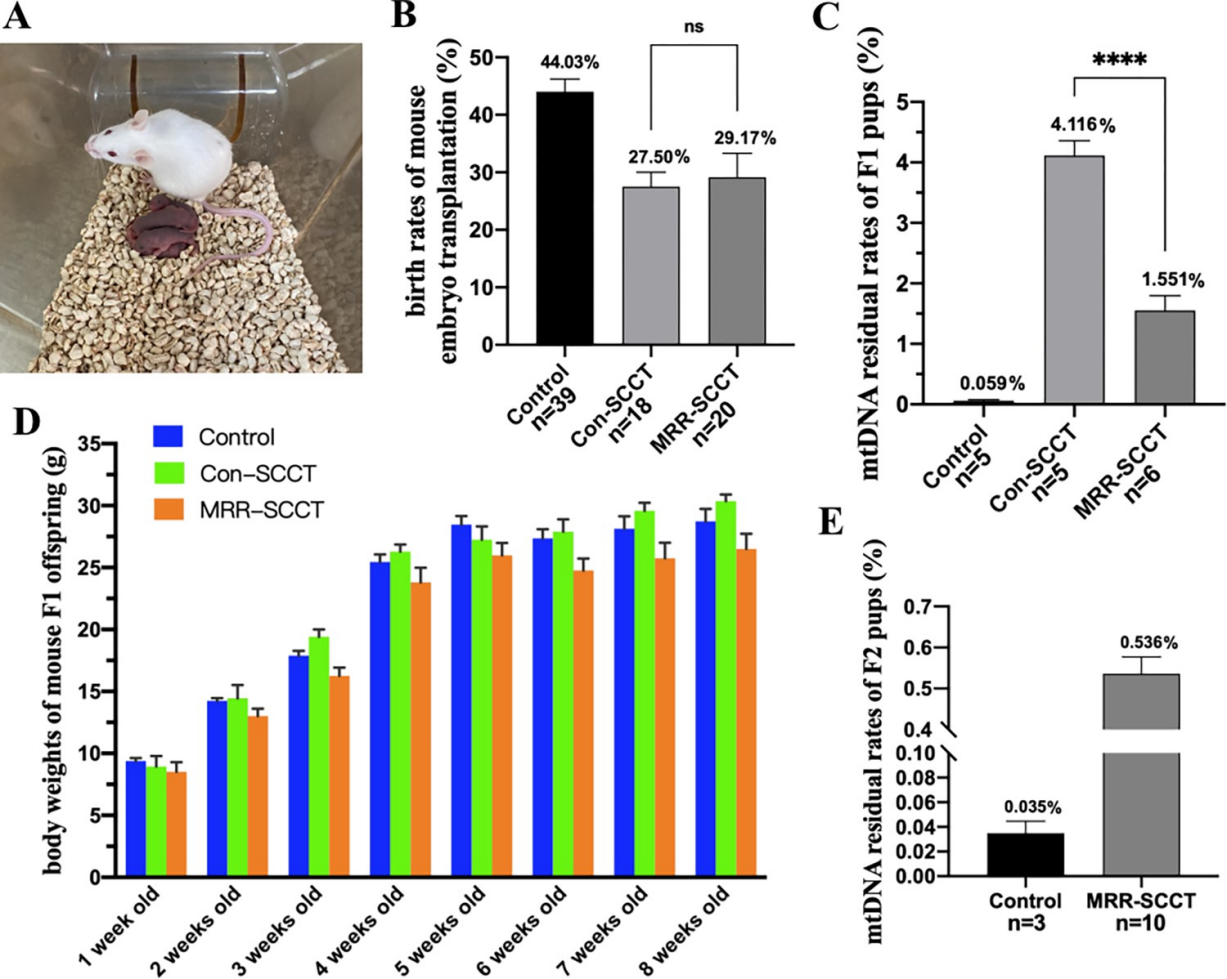
Developmental competence and mtDNA residue in mouse SCCT-generated embryos in vivo. (A) Representative image of F1 live births by reproductive tract transplantation of the MRR-SCCT-generated embryos in vivo. (B) Birth rates of mouse embryo transplantation (%, Y-axis) in the control (*n* = 39), Con-SCCT (*n* = 18), and MRR-SCCT (*n* = 20) groups (X-axis). The error bars represented SEM with mean values shown in each group. “ns” indicated no statistically significant differences between groups (*p* > 0.05, ns: not significant). (C) Quantification of mtDNA residual levels of mouse F1 pups (karyoplast and cytoplasm were from ICR and C57BL/6 mice, respectively, %, Y-axis) in the control (*n* = 5), Con-SCCT (*n* = 5), and MRR-SCCT (*n* = 6) groups (X-axis). The whiskers indicated a significant difference between groups (**** denoted *P* < 0.0001). (D) Body weights of mouse F1 offspring monitored from 1 week to 2 months old in the control, Con-SCCT, and MRR-SCCT groups. (E) Quantification of mtDNA residual levels of mouse F2 pups (mating F1 females in the MRR-SCCT group with wild-type C57BL/6 males, %, Y-axis) in the control (*n* = 3) and MRR-SCCT (*n* = 10) groups (X-axis). Mean values were shown above the columns in both groups. The numerical data were listed in S4 Data. Con-SCCT, conventional SCCT; MRR, maximal residue removal; mtDNA, mitochondrial DNA; SCCT, spindle-chromosomal complex transfer.

Then, we tested developmental competence and mitochondrial residue in vivo by transplanting SCCT-treated embryos at the cleaving 2-cell stage into the reproductive tract of pseudopregnant ICR females. In the MRR-SCCT and Con-SCCT groups, 6 (29.17%) and 5 (27.50%) living F1 pups were delivered out of 20 and 18 transferred embryos, respectively (*p* > 0.05) (Fig 5B), which were born healthy and respired normally (Fig 5A). Toes of pups were biopsied to detect mtDNA heteroplasmy. And results showed that the MRR-SCCT-generated F1 pups had an mtDNA residue of 1.551% on average, which was significantly lower than that in the Con-SCCT group (4.116% on average) (*p* < 0.0001) (Fig 5C). In addition, body weights of F1 pups in the 3 groups monitored from 1 week to 2 months old were all within the normal range for mice at each growth stage (Fig 5D), and there was no statistically significant difference between the MRR or Con-SCCT and control groups (S3 Fig). Furthermore, we mated F1 females in the MRR-SCCT group with wild-type C57BL/6 males and obtained F2 cubs with further lower mtDNA residue (0.536% on average, *n* = 10, Fig 5E) compared to the MRR-SCCT-generated F1 cubs. Overall, these results demonstrated that the reconstructed embryos created by MRR-SCCT were suitable for onward development in vivo and capable of producing living offspring, which had reproductive capacity and were free from mitochondrial genetic drift.

### Mouse ESCs derivation and mtDNA transmission analysis

**Fig 6:**
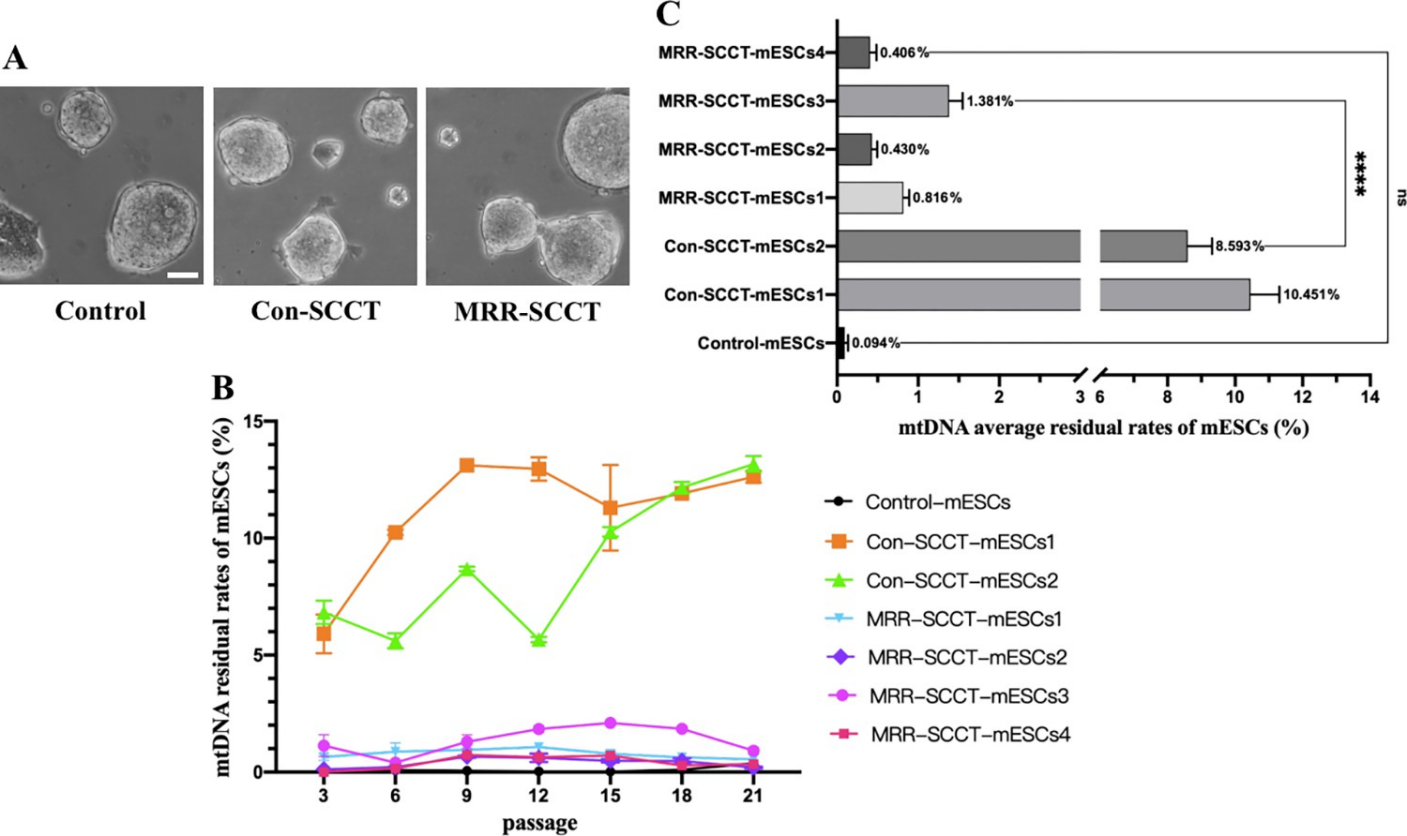
Developmental potential and mtDNA genetic drift analysis in mouse SCCT-generated embryos in vitro. (A) Morphological images of derived mouse ESCs in the control, Con-SCCT, and MRR-SCCT groups. Scale bars, 50 μm. (B) MtDNA residual levels in mouse ES cell lines (%, Y-axis) during 21 passages in the control, Con-SCCT, and MRR-SCCT groups (X-axis). The error bars represented SEM with mean values shown in each group. (C) MtDNA mean residue levels in mouse ES cell lines (%, Y-axis) in the control, Con-SCCT, and MRR-SCCT groups (X-axis). The error bars represented SEM with mean values shown in each group. The whiskers indicated a significant difference between groups (**** denoted *P* < 0.0001). “ns” indicated no statistically significant difference between groups (*p* > 0.05, ns: not significant). The numerical data were listed in S5 Data. Con-SCCT, conventional SCCT; mtDNA, mitochondrial DNA; MRR, maximal residue removal; SCCT, spindle-chromosomal complex transfer.

To further address the concern of genetic drift and support the evaluation of developmental potential, we derived mouse ES cell lines from SCC transferred blastocysts and checked whether the residue level of mtDNA remained stable in following generations. Four and 2 ES cell lines (MRR-SCCT-mESCs1 to 4, and Con-SCCT-mESCs1 to 2) were respectively established from the MRR-SCCT and Con-SCCT-generated blastocysts developed from normally fertilized zygotes, which displayed normal morphology and were indistinguishable from the controls (Fig 6A). For the MRR-SCCT-mESCs, mtDNA residue rates in the 4 lines were similar and stable (range of mean values 0.406% to 1.381%) over 21 generations, which were significantly lower than those in the Con-SCCT-mESCs group (range of mean values 8.593% to 10.451%) (*p* < 0.0001) and similar to the non-manipulated controls (*p* > 0.05) (Fig 6B and 6C). In contrast, mtDNA transmission in the Con-SCCT-mESCs generally displayed an obvious fluctuation and a rising trend (6.368% at passage 3 to 12.896% at passage 21) (Fig 6B). Overall, these results confirmed that the MRR-SCCT could strongly reduce the transmission of spindle-donor mtDNA to the lowest level at a stable state in mESCs, which was consistent with the above showed result that MRR-SCCT-produced offspring did not occur mtDNA genetic drift.

### MtDNA replacement in human oocytes with MRR-SCCT procedure

Finally, the MRR procedure was performed in human MII oocytes. The quantity of mitochondria remained constant without mtDNA replication during the early stages of embryonic development, and investigations in the mouse model showed that the mtDNA proportions of SCC karyoplasts in oocytes and the levels of heteroplasmy in reconstructed oocytes and blastocycles were generally consistent in groups (Figs 2B, 2C, and 4A). Thus in the human model, we utilized mtDNA copy numbers of spindle karyoplasts to measure the level of mtDNA carryover using the MRR technique, which was more convenient and efficient without involving specific mtDNA probes of different types.

**Fig 7:**
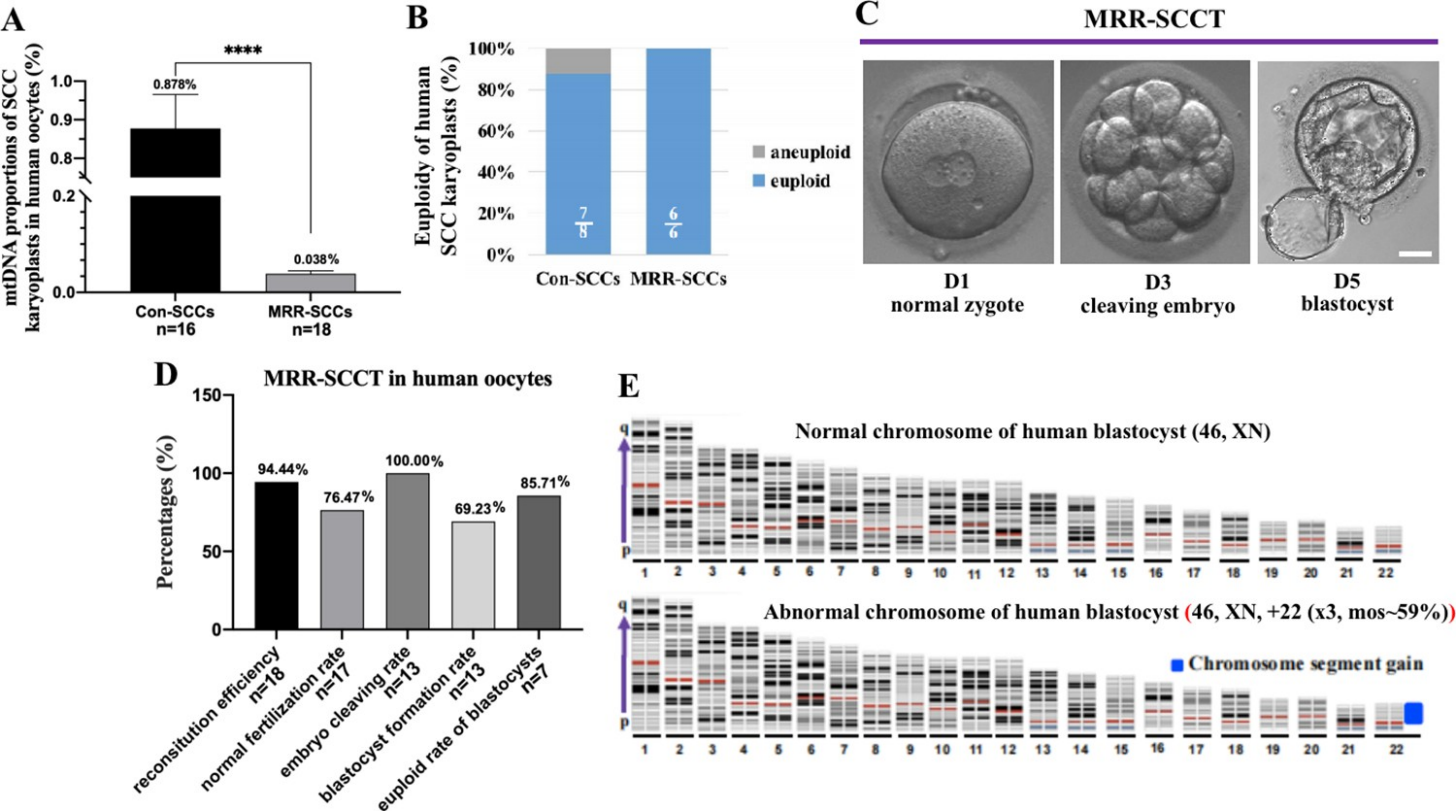
MtDNA replacement in human oocytes with MRR-SCCT procedure. (A) MtDNA proportions of SCC karyoplasts in corresponding enucleated human oocytes (mtDNA copy number of karyoplast/total mtDNA copy numbers of karyoplast and enucleated oocyte, %, Y-axis) in the Con-SCCT (*n* = 16) and MRR-SCCT (*n* = 18) groups (X-axis). The error bars represented SEM with mean values shown in each group. The whiskers indicated a significant difference between groups (**** denoted *P* < 0.0001). (B) Euploidy in human Con-SCC and MRR-SCC karyoplasts. Proportions of euploid (blue, white fractional numbers) and aneuploid (gray) chromosomes were shown in both groups. (C) Morphological images of reconstructed human MRR-SCCT oocyte at the normal zygote, cleaving embryo, and blastocyst stages. The day of fertilization was calculated as Day 0 (D0). Scale bars, 35 μm. (D) Percentages (%, Y-axis) of reconstruction efficiency (*n* = 18), normal fertilization rate (*n* = 17), embryo cleaving rate (*n* = 13), blastocyst formation rate (*n* = 13), and euploid rate (*n* = 7) (X-axis) in human MRR-SCCT-generated blastocysts. Percentage values were shown above the columns. (E) Representative images of normal and abnormal chromosomes in human MRR-SCCT-generated blastocysts. The numbers under chromosomes showed the sequence of paired chromosomes, and the blue chromosome segment indicated an abnormal chromosome. The numerical data were listed in S6 Data. Con-SCCT, conventional SCCT; MRR, maximal residue removal; mtDNA, mitochondrial DNA; SCCT, spindle-chromosomal complex transfer.

By using micropipettes with ID of 10 μm to suck human SCCs and removing any carried cytoplasm through a swinging manipulation (S4A Fig), the karyoplasts exhibited minimal mtDNA proportions in each oocyte (0.038% on average, range 0.011% to 0.092%, *n* = 18), which was remarkably lower than that in the Con-SCCT group (mean 0.878%, *n* = 16) (*p* < 0.0001) (Fig 7A). And euploidy of SCC karyoplasts between the 2 groups were comparable by chromosomal analysis (Fig 7B), suggesting that the MRR manipulation not only could hugely decrease mitochondrial carryovers but also did not damage chromosomes in SCC karyoplasts.

We then reconstructured oocytes with the MRR-SCCT scheme to further evaluate its feasibility in human oocytes, and 17 (94.44%) out of 18 reconstructured oocytes were successfully produced after MRR-SCCT (S4B Fig). The normal fertilization rate of reconstructured oocytes was 76.47%, and all the embryos cleaved and 69.23% developed into blastocysts (Fig 7C and 7D), which were comparable to those previously reported for non-manipulated ICSI-performed human oocytes [13]. Chromosomal CNV analysis showed that 6 out of 7 MRR-SCCT-generated blastocysts contained a diploid genome without numerical or structural abnormality (Fig 7D and 7E). These suggested that the MRR-SCCT procedure was an effective scheme resulting in a nearly negligible carryover of spindle-derived mitochondria and excellent embryo development in human oocytes, which was also consistent with the above showed results in mice.

## Discussion

Inherited mtDNA variant transmission was complex due to the genetic drift and threshold effect. Typically, when the mutation load was greater than 60%, clinical symptoms would appear in most patients with mtDNA diseases [4]. In a previous study of spindle transfer, a significant increase of spindle-donor mtDNA carryover was detected in primate oocytes, implying that heteroplasmic mtDNA might undergo aberrant amplification [11]. Thus, we believed that MR should decrease original mtDNA carryover to the least level, which would most likely prevent detrimental genetic drift. Usually, SCCT would inevitably result in an introduction of certain amounts of mtDNA surrounding the spindle into enucleated recipient oocytes during micromanipulation. Therefore, current technology of spindle transfer required optimization to ultimately remove the residual cytoplasm in SCC karyoplasts.

To this end, here we performed a novel approach called MRR (Fig 1 and S1 Video). As we know, micropipettes with an ID of 15 to 20 μm were usually utilized for SCC isolation in mouse or human oocytes [12,15], which was a routine practice that did not cause SCC damage but resulted in significant cytoplasmic carryovers. According to the volume of spindle observed under inverted microscope and the need for reducing mitochondrial carryover, we thought of using micropipette with a much finer ID (12 μm for mouse oocytes and 10 μm for human oocytes) to absorb SCCs. In addition, in order to further remove carried cytoplasm, we developed a special cytoplasmic-swinging medium. In view of a viscous feature of PVP and the oolemma-softening capacity of serum and CB, we mixed 10% PVP, SSS, and 2.5 μm CB with 5:3:2 ratio to maximally swing away the residual cytoplasm around spindle in SCC karyoplasts.

In this study with the MRR procedure, spindle-donor mtDNA levels in karyoplasts, reconstructed oocytes and blastocysts both significantly decreased to approximately one-sixth of those produced by the conventional approach in mice (*p* < 0.0001) (Figs 2B, 2C, and 4A). Moreover, the mtDNA residue in F1 live births of mice also greatly declined after MRR-SCCT and even much lower in the F2 cubs with mean 0.536% heteroplasmy (Fig 5C and 5E). MtDNA residue in the MRR-SCCT-generated mESCs also exhibited dramatically lower levels compared to that in the Con-SCCT group (*p* < 0.0001) and no statistical difference between with the intact control group (*p* > 0.05) (Fig 6C). Interestingly, the MRR-SCCT mESCs displayed a persistently low and stable transmission of heteroplasmic mtDNA throughout 21 generations, whereas the mtDNA landscape of Con-SCCT mESCs behaved an obvious fluctuation and an increasing trend in total (Fig 6B), strongly supporting our initial assumption that the more originally carried mitochondria remained, the higher odds genetic drift would occur. Importantly, spindle-donor mtDNA carryover lowered to mean 0.038% through the MRR manipulation in human SCC karyoplasts (Fig 7A), representing a maximal carried-mtDNA removal compared to those in the Con-SCC procedure (*p* < 0.0001) and previously reported data [12–16]. Overall, these results indicated that the MRR-SCCT scheme could result in a least amount of spindle-associated mitochondrial carryover and produce offspring without mtDNA genetic drift in vitro and vivo.

The spindle-chromosomal complex in MII oocytes was a relatively sensitive structure that might be perturbed by environmental temperature, chemical reagent, or physical manipulation [13,22]. Concerns were raised that whether the MRR-SCCT method affect spindle-chromosomal complex or not. Our results showed no statistically significant differences in the spindle-chromosomal structure and chromosomal copy numbers between the MRR and Con procedure-generated karyoplasts or reconstructed blastocysts in mice and humans (Figs 2D, 2E, 4C, and 7B). And MRR-SCCT yielded comparable results in the normal fertilization rate and embryo development to those in the Con-SCCT group and intact controls in mice (*p* > 0.05) (Fig 3C–3E), particularly the blastocyte formation rate reached 69.23% on average in human MRR-SCCT-generated embryos (Fig 7D). Following-up postnatal investigations such as body weight (Fig 5D) and pregnant competence in F1 mice produced by MRR-SCCT further provided convincing evidences that mtDNA replacement along with the MRR manipulation was compatible with normal embryo development. Also, MRR-SCCT-derived mESCs contained an almost identical morphology to those in controls and well subcultured throughout 21 generations (Fig 6A). Thus, we concluded that the MRR technique seemed unlikely to cause spindle-chromosomal defects.

In addition, we analyzed that much lower CB concentration and immediate karyoplast fusion after MRR manipulation might contribute to the unexpected satisfying development potential of MRR-SCCT-initiated embryos in this study. To our knowledge, CB known as a microfilament inhibitor could interrupt the PB2 extrusion in oocytes and subsequent embryo development [23,24]. Usually, 7.5 or 10 μm CB was adopted for facilitating micromanipulation during the spindle transfer [12–16]. In consideration of the adverse impact of CB residue even though thoroughly rinsing after transfer, we employed 2.5 μm as the working concentration of CB in this study and manipulated smoothly without oocyte shrinking or sacrifice (S1 Video). Furthermore, we immediately performed HVJ-E protein-assisted fusion after MRR, which was probably favorable to maintain the integraty of spindle and chromosome although minimal remaining cytoplasm in karyoplasts.

During oocyte reconstruction, we first skillfully designed a scheme that reversed the order between ICSI and spindle transfer. In the usual protocol, denucleated oocytes were fused with isolated spindles, transferred to G1 PLUS medium, and recovered approximately 1 h before fertilization via ICSI. However, it had been reported that due to the unique and sensitive biological characteristics of MII oocytes, an influx of extracellular calcium caused by mechanical or chemical manipulations could potentially induce premature activation of oocytes and resumption of meiosis without sperm entering [25,26]. Therefore, it appeared that oocytes experienced SCC aspiration and fusion were prone to trigger spontaneous meiosis resumption during the 1 to 2 h recovery period before ICSI. To solve this problem, we first performed oocyte fertilization by ICSI and then immediately transferred spindle. Results showed that its SCCs morphologically maintained uniform metaphase II stage without premature activation (S1B Fig), contributing to a significant improvement in the normal fertilization rate of reconstructed oocytes compared to that in the transfer-then-ICSI group (*p* < 0.05) (S1A Fig). Meanwhile, we found that the ICSI-then-transfer scheme efficiently shortened SCCT procedure duration without time-spending for oocytes recovery. Moreover, due to the fragile oolemma at fusion region and CB exposure during spindle transfer, certain reconstructed oocytes could not survive subsequent ICSI involving mechanical needle piercing, even though spending a more extended incubation time before ICSI. Therefore, we believed that the ICSI-then-transfer scheme might be conducive to prevent oocyte sacrifice during SCCT.

An MRR-SCC karyoplast with an extremely low proportion of mtDNA (average 0.038%) was absorbed in a human oocyte, implying minimal carryover of mitochondria in SCCT. This achievement has the potential to significantly reduce the incidence of genetic drift. However, we have not been able to conclude yet that this MRR-SCCT procedure is adequately safe in the clinic for preventing the transmission of inherited mtDNA diseases. In human SCCT-generated ESCs, the proportions of maternal mtDNA were found to increase significantly, reaching 53.2% as reported by Yamada and colleagues [15] and even 100% according to Kang and colleagues [16]. Particularly, a recent pilot study applying SCCT for infertility treatment reported for the first time that mtDNA reversal also occurred in humans in vivo. It was revealed that in 1 child produced by SCCT, the maternal mtDNA carryover, initially at a low level (0.8%) at the blastocyst stage, dramatically raised from 30% to 60% in various tissues at birth [27]. In addition, this reversal was also observed in a human ESC line that was derived from a blastocyst produced through pronuclear transfer. This ESC line exhibited an upward drift in heteroplasmy levels by passage 12 [28]. And the mechanisms causing the mtDNA reversal remain unclear. Therefore, we believe that more investigations involving human ESCs will be necessary before the clinical translation of current MRR-SCCT strategy.

Pioneering studies addressing effectiveness and safety of MR in mouse or human model were critical for future clinical application [29]. As we showed here, the MRR-SCCT strategy introducing several technical modifications maximally removed the residue of donor mtDNA, thereafter potentially avoided genetic drift in live births and ESCs of mice, and the well-developed reconstructed embryos confirmed that this procedure might represent a reliable therapy approach to prevent inherited mtDNA diseases.

## Supporting information

S1 Fig The optimization of manipulation order between ICSI and SCC transfer. (A) Normal fertilization rates of mouse reconstructed oocytes (%, Y-axis) in the transfer-then-ICSI (*n* = 50) and ICSI-then-transfer (*n* = 47) groups (X-axis). The error bars represented SEM with mean values shown in each group. The whiskers indicated a significant difference between groups (* denoted *p* < 0.05). The numerical data were listed in S1 Data. (B) Morphological images of spindle and meiotic stage by immunofluorescence labeling with antibody to ɑ-tubulin (green) and DAPI (blue) in the mouse transfer-then-ICSI and ICSI-then-transfer oocytes. BF referred to bright field. Images on the right were higher magnification views of whitely boxed areas in the left. Scale bars, 50 μm.
(TIF)

S2 Fig Fusion of mouse SCC karyoplasts with enucleated oocytes. Representative images of mouse enucleated oocytes before and after fusion with SCC karyoplasts in the Con-SCCT and MRR-SCCT groups. The white arrows indicated SCC karyoplasts. Scale bars, 20 μm.
(TIF)

S3 Fig Body weights of 8-week-old mouse F1 offspring. Body weights of mouse F1 offspring monitored at the 8 weeks old in the control (*n* = 5), Con-SCCT (*n* = 5), and MRR-SCCT (*n* = 5) groups. The error bars represented SEM with mean values shown in each group. “ns” indicated no statistically significant differences between groups (*p* > 0.05, ns: not significant). The numerical data were listed in S4 Data.
(TIF)

S4 Fig Human SCC karyoplasts and fusion with enucleated oocytes. (A) Representative images of human Con-SCCT and MRR-SCCT karyoplasts isolated by micropipettes with ID of 15 μm and 10 μm, respectively. Scale bars, 20 μm. (B) Representative images of human enucleated oocytes before and after fusion with MRR-SCC karyoplasts. White arrows indicated the spindle. Scale bars, 35 μm.
(TIF)

S1 Table Sequences of primer and probe for detecting mtDNA copy number by ddPCR. (DOCX)

S1 Video Micromanipulation of MRR-SCCT procedure in mouse MII oocytes. (MP4)

S1 Data The individual numerical values in Figs 2B, 2C, 2E, and S1A. (XLSX)

S2 Data The individual numerical values in Fig 3A, 3C, 3D, and 3E. (XLSX)

S3 Data The individual numerical values in Fig 4A and 4C. (XLSX)

S4 Data The individual numerical values in Figs 5B–5E and S3. (XLSX)

S5 Data The individual numerical values in Fig 6B and 6C. (XLSX)

S6 Data The individual numerical values in Fig 7A, 7B, and 7D. (XLSX)

## Abbreviations

BSA: bovine serum albumin
CNV: copy number variation
COC: cumulus-oocyte complex
Con-SCCT: conventional SCCT
ESC: embryonic stem cell
hCG: human chorionic gonadotrophin
ICSI: intracytoplasmic sperm injection
ID: inner diameter
mHTF: modified human tubal fluid
MR: mitochondrial replacement
MRR: maximal residue removal
mtDNA: mitochondrial DNA
PMSG: pregnant mare serum gonadotrophin
PVP: polyvinylpyrrolidone
SCCT: spindle-chromosomal complex transfer
SSS: serum substitute supplement
