# Supplementary figures and images for: Significant decrease of maternal mitochondria carryover using optimized spindle-chromosomal complex transfer
Source: PLoS Biol. 2023 Oct 5;21(10):e3002313. doi: 10.1371/journal.pbio.3002313 (PMC10553349; doi:10.1371/journal.pbio.3002313)

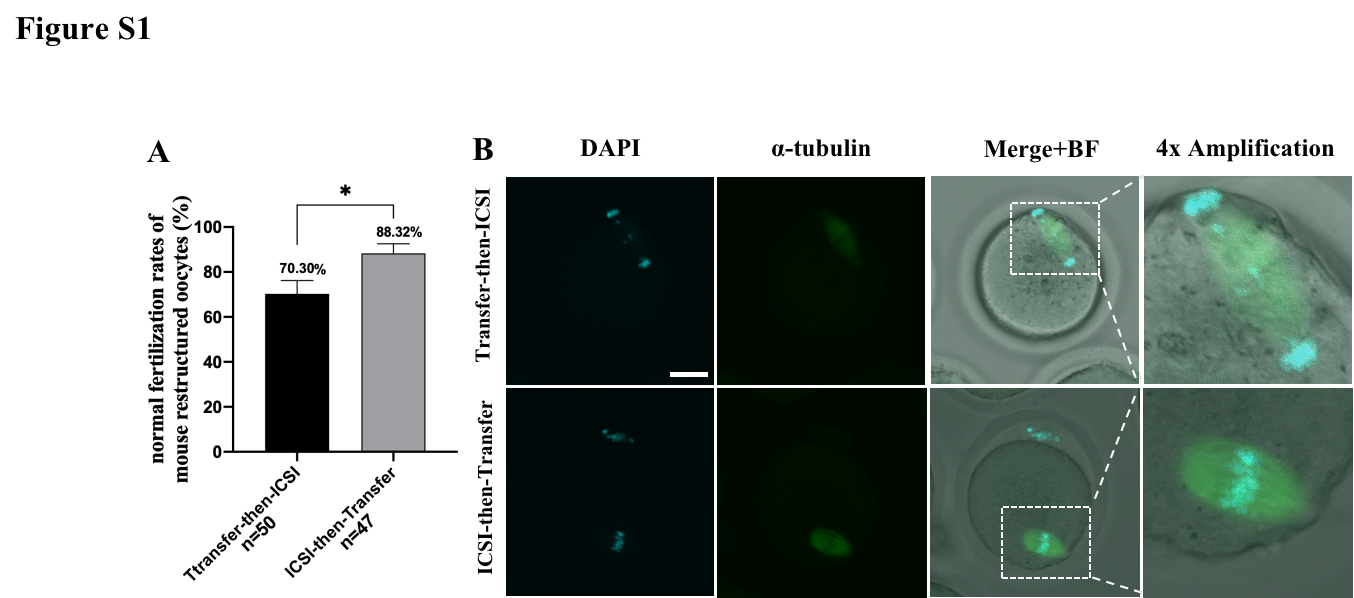

Supplement: S1 Fig — (A) Normal fertilization rates of mouse reconstructed oocytes (%, Y-axis) in the transfer-then-ICSI (n = 50) and ICSI-then-transfer (n = 47) groups (X-axis). The error bars represented SEM with mean values shown in each group. The whiskers indicated a significant difference between groups (* denoted p < 0.05). The numerical data were listed in S1 Data. (B) Morphological images of spindle and meiotic stage by immunofluorescence labeling with antibody to ɑ-tubulin (green) and DAPI (blue) in the mouse transfer-then-ICSI and ICSI-then-transfer oocytes. BF referred to bright field. Images on the right were higher magnification views of whitely boxed areas in the left. Scale bars, 50 μm. (TIF) [file pbio.3002313.s001.tif]

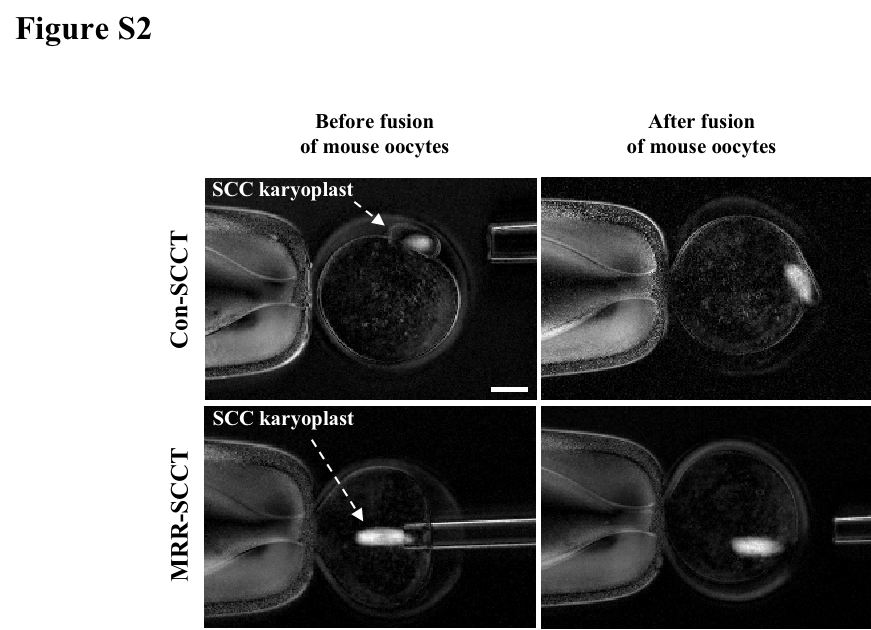

Supplement: S2 Fig — Representative images of mouse enucleated oocytes before and after fusion with SCC karyoplasts in the Con-SCCT and MRR-SCCT groups. The white arrows indicated SCC karyoplasts. Scale bars, 20 μm. (TIF) [file pbio.3002313.s002.tif]

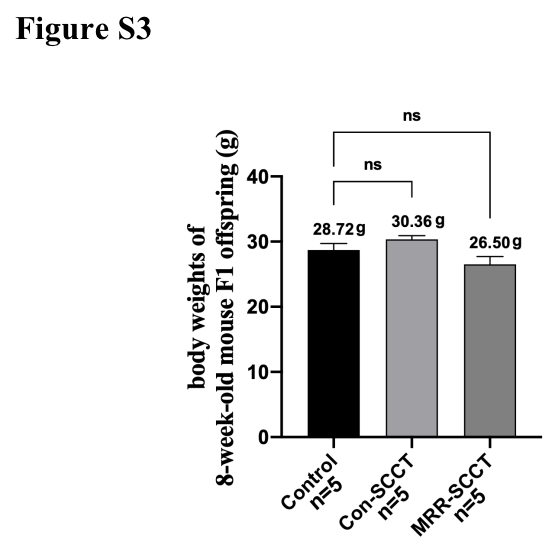

Supplement: S3 Fig — Body weights of mouse F1 offspring monitored at the 8 weeks old in the control (n = 5), Con-SCCT (n = 5), and MRR-SCCT (n = 5) groups. The error bars represented SEM with mean values shown in each group. “ns” indicated no statistically significant differences between groups (p > 0.05, ns: not significant). The numerical data were listed in S4 Data. (TIF) [file pbio.3002313.s003.tif]

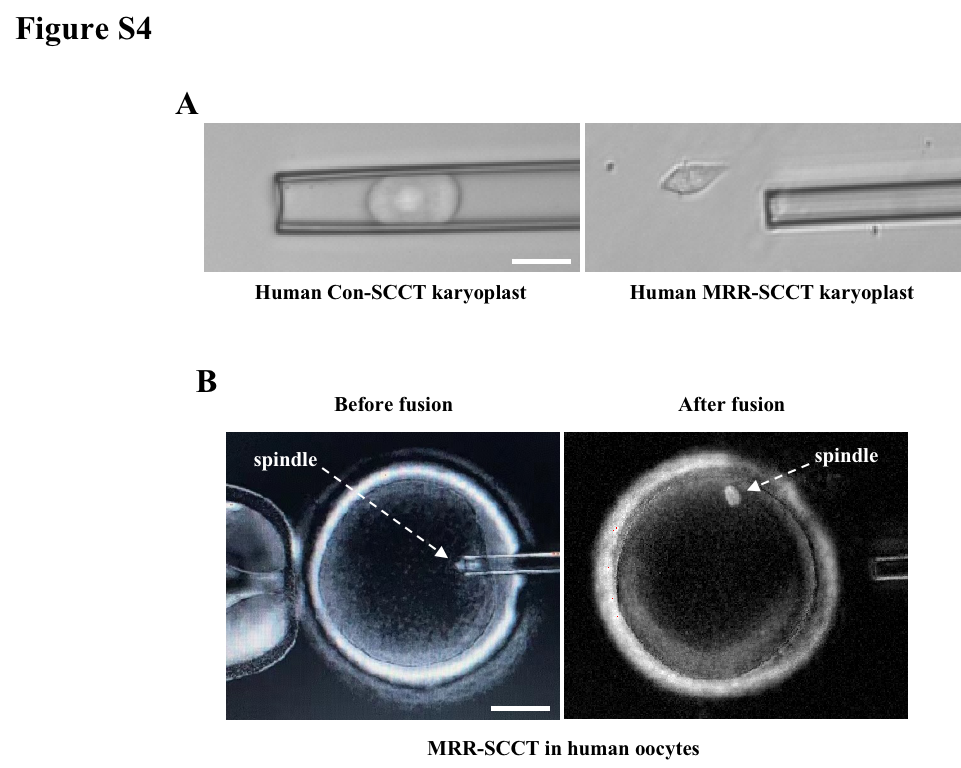

Supplement: S4 Fig — (A) Representative images of human Con-SCCT and MRR-SCCT karyoplasts isolated by micropipettes with ID of 15 μm and 10 μm, respectively. Scale bars, 20 μm. (B) Representative images of human enucleated oocytes before and after fusion with MRR-SCC karyoplasts. White arrows indicated the spindle. Scale bars, 35 μm. (TIF) [file pbio.3002313.s004.tif]
