# Supplementary material for: Significant decrease of maternal mitochondria carryover using optimized spindle-chromosomal complex transfer
Source: PLoS Biol. 2023 Oct 5;21(10):e3002313. doi: 10.1371/journal.pbio.3002313 (PMC10553349; doi:10.1371/journal.pbio.3002313)
Supplement: S1 Table — (DOCX) [file pbio.3002313.s005.docx]

**Table S1. Sequences of primer and probe for detecting mtDNA copy number by ddPCR.**

| **Name** | **Species** | **Strain** | **Sequence** |
| --- | --- | --- | --- |
| mPrimer_F | Mouse | NA | TTCTGAATAAACCCAGAAGAGAG |
| mPrimer_R | Mouse | NA | CAACTAGAATTAGCGTTAGGGATA |
| mProbe_HEX | Mouse | ICR | TGTA+C+AGGT+T+G+AT |
| mProbe_FAM | Mouse | C57BL/6 | AGTGTA+C+A+GGT+T+A+AT |

NA: not applicable.
